# Development of a dual hybrid AAV vector for endothelial-targeted expression of von Willebrand factor

**DOI:** 10.1038/s41434-020-00218-6

**Published:** 2021-01-17

**Authors:** Elena Barbon, Charlotte Kawecki, Solenne Marmier, Aboud Sakkal, Fanny Collaud, Severine Charles, Giuseppe Ronzitti, Caterina Casari, Olivier D. Christophe, Cécile V. Denis, Peter J. Lenting, Federico Mingozzi

**Affiliations:** 1 Généthon, 91000 Evry, France; 2 Université Paris-Saclay, Université Evry, INSERM, Généthon, Integrare research unit UMR_S951, 91000 Evry, France; 3 Laboratory of Hemostasis, Inflammation and Thrombosis, Institut National de la Santé et de la Recherche Médicale UMR_1176, Université Paris-Saclay, 94276 Le Kremlin-Bicêtre, France; 4 Present Address: Spark Therapeutics, Philadelphia, PA 19103 USA

**Keywords:** Molecular biology, Diseases

## Abstract

Von Willebrand disease (VWD), the most common inherited bleeding disorder in humans, is caused by quantitative or qualitative defects in von Willebrand factor (VWF). VWD represents a potential target for gene therapy applications, as a single treatment could potentially result in a long-term correction of the disease. In recent years, several liver-directed gene therapy approaches have been exploited for VWD, but their efficacy was generally limited by the large size of the VWF transgene and the reduced hemostatic activity of the protein produced from hepatocytes. In this context, we aimed at developing a gene therapy strategy for gene delivery into endothelial cells, the natural site of biosynthesis of VWF. We optimized an endothelial-specific dual hybrid AAV vector, in which the large VWF cDNA was put under the control of an endothelial promoter and correctly reconstituted upon cell transduction by a combination of *trans-*splicing and homologous recombination mechanisms. In addition, we modified the AAV vector capsid by introducing an endothelial-targeting peptide to improve the efficiency for endothelial-directed gene transfer. This vector platform allowed the reconstitution of full-length VWF transgene both in vitro in human umbilical vein endothelial cells and in vivo in VWD mice, resulting in long-term expression of VWF.

## Introduction

Von Willebrand factor (VWF) is a large multimeric glycoprotein circulating in blood, where it exerts fundamental hemostatic functions, such as serving as carrier of coagulation factor VIII (FVIII), interacting with collagen and mediating platelet adhesion at wound sites [1–5]. Quantitative, structural, or functional abnormalities of VWF lead to von Willebrand disease (VWD), a bleeding disorder with a prevalence of up to 1% in the general population, making it the most common genetic coagulation disorder [6]. The clinical manifestations of VWD are bleeding symptoms that can vary depending on the underlying molecular defect and the extent of the deficiency [7, 8]. The current treatment of VWD is based on the on-demand administration of desmopressin, plasma-derived or recombinant VWF concentrates [9–11]. Although current therapies are satisfactory for a large percentage of patients, there are still limitations mostly related to the short-term efficacy of these treatments [12]. Consequently, the quality of life of VWF patients is negatively affected, particularly for type 3 VWD patients who experience a severe bleeding phenotype and may require up to two to three times weekly intravenous infusions to manage their symptoms [13]. VWD represents a potential target for gene therapy approaches, as a single treatment could potentially result in a long-term correction of the disease and even a partial correction could be beneficial in lowering the bleeding risks, like in the case of hemophilia [14]. In recent years, both ex vivo and in vivo gene therapy strategies to treat VWD have been investigated, trying to overcome limitations related to the large size of the VWF cDNA (8.4 kb) and the VWF transient expression [15–19]. However, to date only a partial correction of VWD disease phenotype was achieved in vivo in a VWD mouse model [20] by expressing VWF from the liver [17–19]. Indeed, while VWF expressed from hepatocytes showed hemostatic capacity in the early phases after gene transfer, this was gradually lost over time with the lack of an efficient VWF multimerization [19]. In fact, the complex VWF biosynthesis occurs physiologically in megakaryocytes and endothelium, where protein monomers are arranged into dimers in the endoplasmic reticulum and into multimers in the Golgi apparatus [5]. These multimers, whose size strongly influences the hemostatic potential of VWF, are secreted constitutively at basal level or stored in specialized organelles and released upon endothelial stimulation [1]. The differences in hepatocytes and endothelial cells (ECs) cell machinery and function could therefore explain the limited hemostatic capacity of hepatocyte derived VWF. Being the natural site of VWF production, ECs represent, therefore, an ideal target for an in vivo directed gene therapy for VWD. Here, we exploited a dual hybrid AAV, a platform based on a *trans-*splicing vector provided with a highly recombinogenic region to enhance the recombination rate between the two AAVs [21–23], to express the large VWF transgene in the endothelium. The dual hybrid AAV strategy has already demonstrated to be a promising tool to overcome the AAV limited cargo capacity [22, 24] and to allow an efficient gene transfer and reconstitution of full-length therapeutic proteins [23, 25–28]. Accordingly, we developed a dual hybrid expression cassette in which the coding sequence (CDS) of VWF was split in two halves and put under the control of the intercellular adhesion molecule 2 (ICAM2) promoter [29], thus guaranteeing the transgene expression specifically from ECs. In order to improve the gene delivery into this cell type, we optimized an AAV9 by expressing a selected endothelial-targeting peptide on the vector capsid site A589 [30] via insertional mutagenesis. The optimized dual hybrid AAV9 vector was able to efficiently transduce human umbilical vein ECs (HUVECs) and to reconstitute full-length VWF both in vitro and in the VWD mouse model.

## Materials and methods

### Generation of VWF and luciferase expression cassettes

The VWF cDNA sequences used in the study were synthetized by GeneArt (Thermo Fisher Scientific). Two separate AAV vector plasmids (5′ VWF and 3′ VWF) were employed to generate dual hybrid AAV vectors and contained either the promoter followed by the N-terminal portion of the codon optimized VWF transgene (5′ VWF) or the C-terminal portion of the transgene CDS followed by the polyA signal (3′ VWF) (Table 1). The entire expression cassette was flanked by the inverted terminal repeats of AAV serotype 2 for vector packaging. The VWF CDS in 5′ VWF plasmid was put under the control of a previously described minimal human ICAM2 promoter [29] or a human antitrypsin promoter [31]. The firefly luciferase expression cassette was put under the control of a ubiquitous mouse 3-phosphoglycerate kinase promoter [32]. The details of cloning strategies as well as primers and plasmid sequences are available upon request.

**Table 1:** The size of the elements of the expression cassette used to produce the dual hybrid AAVs.

Vector name	Expression cassette elements	Sequence size (bp)
AAV-5′ VWF	AAV2 5′ + 3′ ITRs	260
	ICAM2 promoter	336
	5′ VWF coding sequence	4059
	Splicing donor site (SD)	82
	AK homology region	77
AAV-3′ VWF	AAV2 5′ + 3′ ITRs	260
	AK homology region	77
	Splicing acceptor site (SA)	51
	3′ VWF coding sequence	4383
	Minimal polyadenylation signal (pA)	48

### Generation of AAV9 capsid mutants

A pAAV2_9 rep/cap plasmid used in the triple transfection for AAV production [33] was used as template for in vitro mutagenesis, in order to introduce two incompatible *SfiI* restriction sites in the AAV9-cap open reading frame. The strategy enables to clone oligonucleotides in an oriented fashion, as previously reported [30]. The mutagenesis was performed by a reverse PCR with the forward primer 5′- AAAGATCTGGCCCAGGCGGCCACCGGCTGGGTTCAAAACCA-3′ and the reverse primer 5′- AAAGATCTGGCCTGCTTGGCCACTCTGGTGGTTTGTGGCCA-3′ using the QuikChange II XL Site-Directed Mutagenesis Kit (Agilent). The AAV9mut variants were generated by designing overlapping oligos encoding for each heptapeptide. Once annealed, they were cloned directly into the overhangs generated by restriction digestion of the pAAV2_9 rep/cap plasmid with the *SfiI* enzyme.

### Production of AAV vectors

AAV vectors were prepared as previously described [33]. Briefly, genome-containing vectors were produced in roller bottles following a triple transfection protocol with cesium chloride gradient purification. Titers of AAV vector stocks were determined using real-time qPCR performed in ABI PRISM 7900 HT Sequence Detector using Absolute ROX mix (Taqman, Thermo Fisher Scientific, Waltham, MA) and SDS-PAGE, followed by SYPRO Ruby protein gel stain and band densitometry.

### In vitro studies

HEK293, HuH7, and HUVECs (Lonza) were maintained under 37 °C, 5% CO_2_ condition in Dulbecco’s modified Eagle’s Medium supplemented with 10% FBS, 2 mM GlutaMAX (Thermo Fisher Scientific, Waltham, MA) or endothelial growth medium (EGM-2 BulletKit Medium, Lonza), respectively. None of the cell lines used in the study were authenticated. All cell lined were of commercial origin and were free of mycoplasma contamination. Cells were seeded in 12-well plates (2 × 10^5^ cells/well) and transduced with the dual AAV vectors at different multiplicity of infection (MOI). Seventy-two hours after transfection, cells and conditioned media were harvested and analyzed for VWF expression. DNA was extracted from cell lysates with the QIAgen Blood Core Kit B precipitation method (Qiagen) following the manufacturer’s instruction.

### In vivo studies

VWF deficient [20] and wild-type (wt) mice on a C57BL/6 background were used throughout this study. Mouse studies were performed according to the French and European legislation on animal care and experimentation (2010/63/EU) and approved by the local institutional ethical committee (protocol n.2017-014-B). In the experiments with VWD mice, male mice aged 6–8 weeks were used. For this mouse study, animals were assigned randomly to treatment groups which were composed of five animals per group. In the experiments with wt mice, male mice aged 8 weeks were used. For mouse experiment using wt mice, four mice per group were used. The results obtained by bioimaging were so clear-cut that there was no need to determine sample size to reach statistical significance. Blood samples were collected from the retro-orbital plexus in 3.8% citrate coated capillary tubes (Hirschmann Laborgeräte, Germany). At euthanasia, tissues were collected and snap-frozen for additional studies. Operators in charge of the sample analysis were blinded to the treatment groups. No data were excluded from the analysis.

### VWF detection assays

For western blot analysis, HuH7 and HUVEC protein lysates were prepared using 10 mM PBS (pH7.4) containing 1% of Triton-X100 and protease inhibitors (Roche Diagnosis). Protein concentration was determined using the BCA Protein Assay (Thermo Fisher Scientific). SDS-page electrophoresis was performed in a 4–15% gradient polyacrylamide gel. After transfer, the membrane was blocked with Odyssey buffer (LI-COR Biosciences) and incubated with an anti-VWF antibody (rabbit polyclonal sc-14014, Santa Cruz Biotechnology). The membrane was washed and incubated with the appropriate secondary antibody (LI-COR Biosciences) and visualized by Odyssey Imaging System (LI-COR Biosciences). For western blot quantification, we used the Image Studio Lite Ver 4.0 software. VWF antigen levels were measured using an immunosorbent-assay using polyclonal anti-VWF antibodies as described previously [34]. Pooled murine plasma prepared from 20 (males and females) C57Bl/6 mice was used as reference.

### FVIII activity assay

FVIII Chromogenic activity was determined using the Biophen FVIII assay kit (Hyphen, Neuville-sur-Oise, France) following the manufacturer’s instruction.

### In vivo bioimaging

WT mice were injected with D-luciferin substrate (Perkin Elmer) intraperitoneally (150 µg/g of body weight). After 3–4 min, mice were anesthetized with 2.5% isoflurane and oxygen. Mice were then placed in a light-tight chamber, and images were acquired using an IVIS Spectrum Instrument (Xenogen, Alameda, CA). For each mouse, the images were taken at 10–12 min after the substrate injection. The visual output of the images represents the number of photons emitted/second/cm^2^ as a false color image where the maximum is red and the minimum is dark blue. All animals were imaged at 15 and 35 days post AAV injection.

### Vector genome copy number (VGCN)

DNA from tissues was extracted after whole-organ homogenization using the QIAgen Blood Core Kit B precipitation method (QIAGEN). VGCN was determined using a qPCR assay using 100 ng of DNA. The PGK promoter-specific primers and the probe (forward primer 5′-GCACATTCTTCACGTCCGTT-3′, reverse primer 5′-AGGGTACTAGTGAGACGTGC-3′, probe (FAM) 5′-GAAGGTTCCTTGCGGTTCG-3′ (TAMRA) were synthesized by Thermo Scientific (Waltham, MA, USA). Mouse Titin was used as a normalizing gene in the qPCR assay (forward primer 5’-AAAACGAGCAGTGACGTGAGC-3’, reverse primer 5’-TTCAGTCATGCTGCTAGCGC-3’, probe (VIC) 5’-TGCACGGAAGCGTCTCGTCT CAGTC-3’ (TAMRA) were synthesized by Thermo Scientific, Waltham, MA, USA). For vector copy number determination per cell, PGK copies were normalized on the mouse titin copies multiply for the ploidy of the cells (*n* = 2). Each sample was tested in triplicate.

### Statistical analysis

All the data shown in the present manuscript are reported as mean ± standard deviation (SD). The number of sampled units, *n*, upon which we reported statistic, is the single mouse for the in vivo experiments (one mouse is *n* = 1). Statistical analyses were conducted with GraphPad Prism 7 software (GraphPad Software). For all the data sets, data were analyzed by Student’s *t* test for two-group comparisons or parametric tests (one- and two-way ANOVA with Tukey’s or Dunnett’s post hoc correction for comparisons with more than two groups). *p* values < 0.05 were considered significant. The statistical analysis performed for each data set is depicted in figure legends.

## Results and discussion

**Fig. 1:**
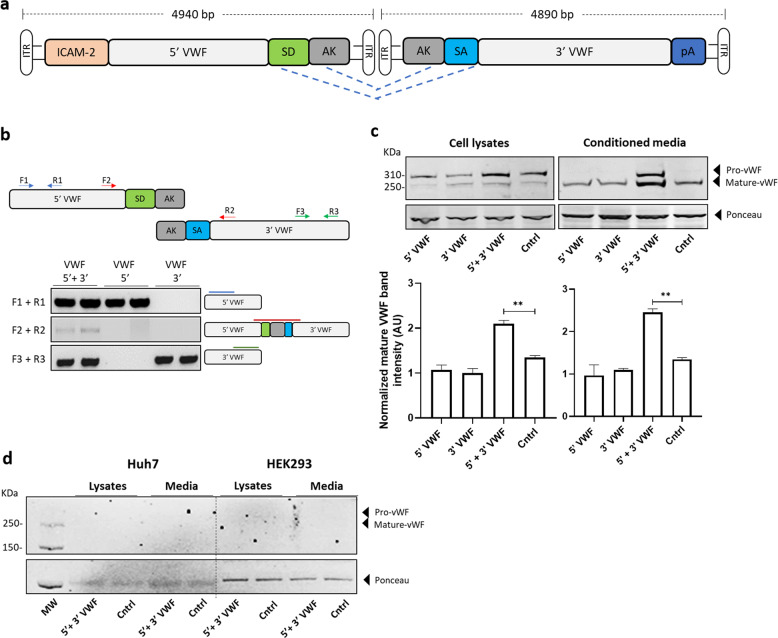
Generation of a dual hybrid AAV vector expressing VWF in HUVEC cells. **a** Schematic representation of the dual hybrid AAV vector. ITR inverted terminal repeats for AAV packaging, ICAM2 intercellular adhesion molecule 2 promoter, 5′ VWF 5′ region of the VWF coding sequence, from nucleotide +1 to +4059, SD splicing donor site, AK recombinogenic region from F1 phage genome, SA splicing acceptor site, 3′ VWF 3′ region of the VWF coding sequence, from nucleotide +4060 to +8442. **b** On the top, scheme of the primers used to amplify VWF coding sequence from vector genome. On the bottom left, PCR analysis of the VWF amplicons. On the bottom right, a schematic representation of the amplified viral genomic region is depicted. **c** Representative western blot analyses on HUVEC cell lysates and conditioned media samples collected 72 h post transduction. On the bottom, histogram reporting the western blot quantification via densitometric analysis. Cntrl, untransduced HUVEC cells. Data are reported as mean ± SD from experiments performed in triplicate, **p < 0.01 (one-way ANOVA). **d** Representative western blot analyses on Huh7 and HEK293 cell lysates and conditioned media samples collected 72 h post transduction. Cntrl, untransduced cells.

**Fig. 2:**
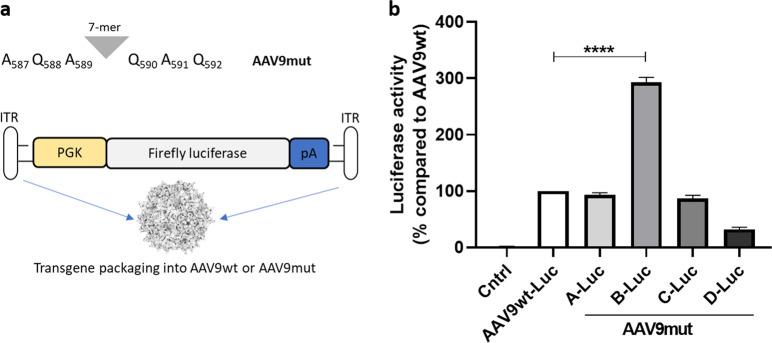
Heptapeptide-modified AAV9 capsids and their transduction efficiencies in HUVECs. **a** Scheme of the heptapeptides (7-mers) inserted at AAV9 capsid site A589 and the incorporated expression cassette consisting in firefly luciferase reporter under the control of the PGK promoter. pA polyA signal, ITR inverted terminal repeats for AAV genome packaging. **b** Luciferase activity in HUVECs expressed as percentage relative to that of AAV9wt. Cntrl, untransduced HUVECs. Data are reported as mean ± SD from experiments performed in triplicate, ****p < 0.001 (unpaired *t*-test).

**Fig. 3:**
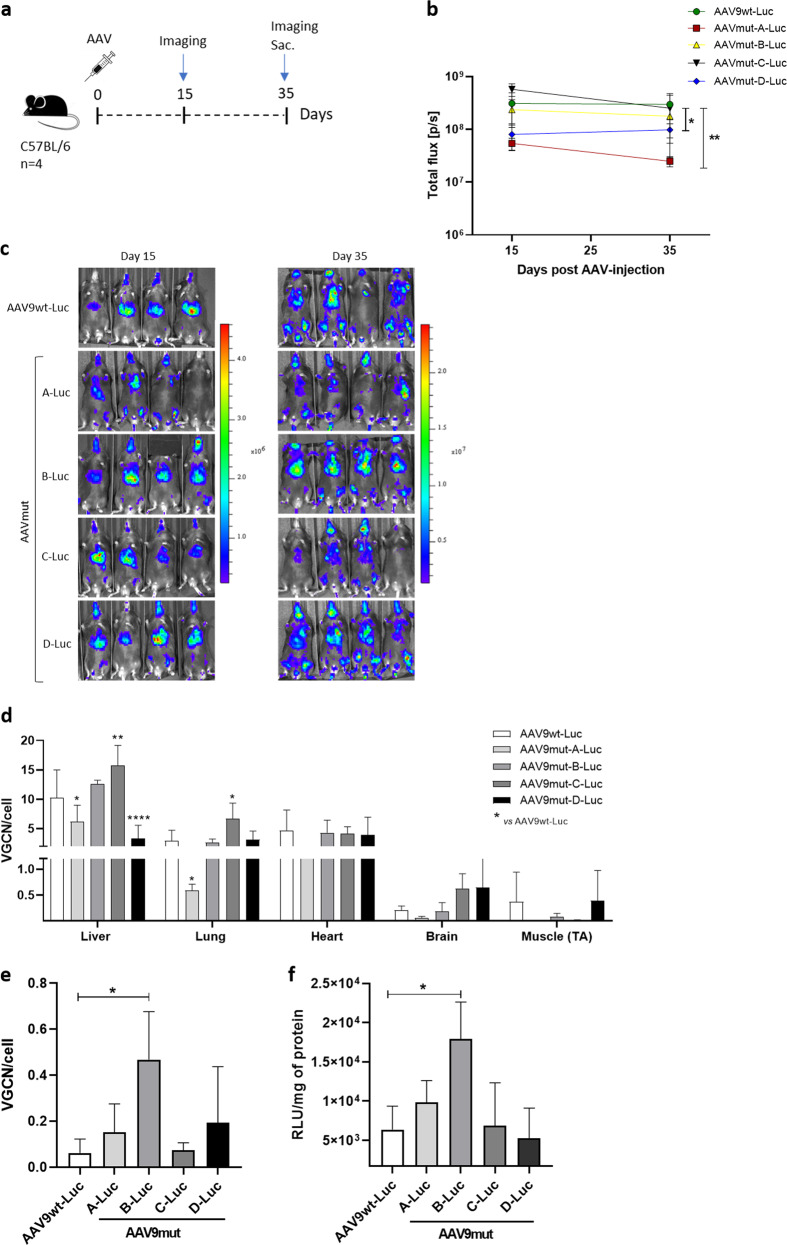
In vivo transgene expression and biodistribution of AAV9 capsid-modified vectors. **a** Scheme of the study design. AAV vectors were injected in C57Bl/6 mice (*n* = 4) at a dose of 2 × 1010 vg/mouse. Blue arrows represent timing of bioimaging. **b** Graph reporting the luciferase reporter expression over time expressed in total flux emitted and recorded by IVIS instrument. p/s photons per second. **c** In vivo luciferase expression in mice injected with AAV9wt and mut variants. The visual output of the images represents the number of photons emitted/second/cm^2^ as a false color image where the maximum is red and the minimum is dark blue. At day 15, the expression range was 250,000–4,600,000 photons/s/cm^2^, and at day 35, the expression range was 1,000,000–25,000,000 photons/s/cm^2^. **d** Vector genome copy number (VGCN) per cell in different organs collected at sacrifice. Data are reported as mean ± SD, *p < 0.05, **p < 0.01, ****p < 0.0001 (two-way ANOVA with Dunnett’s post hoc correction). **e**, **f** VGCN per cell and luciferase activity in aorta. Data are reported as mean ± SD, *p < 0.05 (one-way ANOVA with Dunnett’s post hoc correction).

**Fig. 4:**
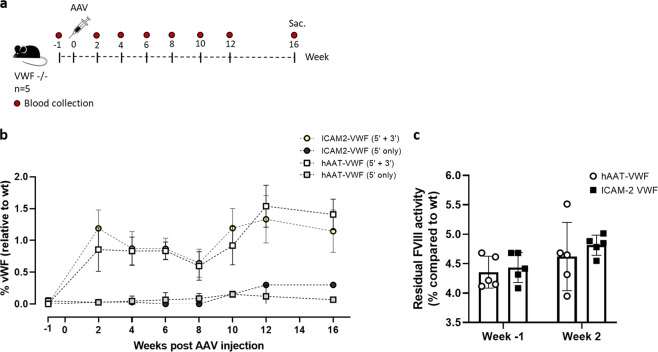
VWF expression in VWD mice administered with endothelial or liver-specific dual hybrid AAV vectors. **a** Scheme of the study design. AAV vectors were injected in VWF deficient mice (VWF^−/−^, *n* = 5) at a dose of 5 × 1011 vg/mouse. Red symbols represent timing of blood collection. Sac sacrifice. **b** Graph reporting VWF expression levels in plasma over time, expressed as percentage relative to the average levels of wt mice (set as 100%). **c** Graph reporting the measurement of FVIII activity (% FVIIIa:C) in plasma samples collected from mice 1 week before and 2 weeks after AAV injection.

We generated a dual hybrid [25] adeno-associated virus vector in which the 8.4 kb VWF CDS was split in two AAV9 vectors (named AAV-5′ VWF and AAV- 3′ VWF) and put under the control of a minimal endothelial-specific ICAM2 promoter [29], thus permitting to obtain two expression cassettes of about 4.9 kb each (Fig. 1a and Table 1). The recombinogenic region consisted of a previously described [25] 77 bp exogenous sequence derived from the F1 phage genome (AK) (Table 1), which was placed downstream the donor splice site (SD) in the AAV-5′ VWF and upstream the acceptor splice site in the AAV-3′ VWF in order to increase the rate of the homologous recombination between the two genomes (Fig. 1a). We tested the efficiency of this dual hybrid AAV to reconstitute the large gene of VWF in vitro by infecting HUVECs at a MOI of 10^3^ of each vector. Seventy-two hours post transduction, we performed DNA extraction and analyzed the viral genome by PCR amplification with primer sets designed to specifically amplify regions of the 5′ VWF, 3′ VWF, or the junction between the two halves of the VWF CDS. We detected the presence of the 5′ VWF and 3′ VWF PCR amplicons (Fig. 1b, blue and green lines) in the cells transduced with AAV-5′ VWF and 3′ VWF, respectively. In the cells transduced with both vectors, we detected the correct PCR amplicon corresponding to the junction region comprising the splicing donor, AK sequence, and splicing acceptor (Fig. 1b, red lines), confirming the reconstitution of the full-length VWF transgene. On the other hand, we did not detect PCR amplicons corresponding to the genomic product containing the AAV ITRs, which is also expected upon recombination between the dual hybrid vector [23], most likely because of an inefficient PCR amplification of these DNA regions with secondary structures [35]. HUVECs do expresses VWF and therefore we analyzed whether protein levels would increase upon transgene expression. Whereas no effect on VWF protein levels upon transduction with 5′-VWF or 3′-VWF alone was observed, an increase in VWF protein was observed for the 5′-VWF/3′-VWF combination, both intra- and extracellularly (2 and 2.5-fold, respectively, Fig. 1c). Interestingly, in the conditioned media from transduced cells we detected the secreted 310-kDa VWF precursor form, which was not present in the control samples (Fig. 1c). In hepatocyte derived Huh7 and renal HEK293 cells, transduced with the dual AAV vector in the same experimental setting as for HUVEC, we did detect the genomic product derived from the correct recombination between the dual hybrid AAVs (Supplementary Fig. 1) but we did not detect any VWF protein expression (Fig. 1d), likely due of the lack of activity of the endothelial promoter ICAM2 in these cell types. To further optimize the vector for a transgene delivery into ECs, we took advantage of a selected endothelium-targeting peptide to be expressed on the surface of the AAV capsid site A589 (VP1 numbering), a strategy which has already been described for AAV2 and AAV9 vector retargeting to various cell lines, including the vasculature [30, 36–40]. Using a similar strategy, we generated wt and mutant AAV9 vectors (AAV9wt and AAV9mut-A, -B, -C, and -D) displaying different endothelium-targeting heptapeptides on the capsid (Fig. 2a). These peptides were previously obtained by in vitro selection of an AAV2 library (unpublished data). To compare the transduction efficiency of the different vectors, they were all produced with an expression cassette consisting in a firefly luciferase reporter gene under the control of the ubiquitous PGK promoter (Fig. 2a). We transduced HUVEC with the vector mutants at MOI of 10^4^ and measured luciferase activity in the cell lysates after 48 h. The AAV9mut-A and -C mutants displayed an activity comparable to the one of cells transduced with AAV9wt, while AAV9mut-D showed a 70% reduced activity compared to the control AAV9wt vector (Fig. 2b). On the other hand, enzyme activity was significantly higher in cells transduced with AAV9mut-B (2.9 ± 0.06-fold, *p* < 0.0001, Fig. 2b), thus indicating an increased HUVEC transduction efficiency of this vector compared to AAV9wt. We then performed a comparative study of AAV9wt and AAV9mut biodistribution and vector-mediated transgene expression following systemic delivery in C57Bl/6wt mice (*n* = 4 per group). To better highlight vector spread throughout the body, the PGK promoter was used to drive the expression of luciferase. After the intravenous injection of the AAV vectors at a dose of 2 × 10^10^ vector genomes (vg)/mouse, animals were imaged at 15 and 35 days post administration (Fig. 3a). All the animals displayed luciferase expression at day 15, which remained sustained over time (Fig. 3b). Total flux measured from the ventral images of the animals at day 35 defined a high expression group comprising AAV9wt (2.9 × 10^8^ photons/s [p/s]), AAV9mut-B (1.7 × 10^8^ p/s), and AAV9mut-C (2.5 × 10^8^ p/s), while AAV9mut-A and -D showed a lower luciferase expression (9 × 10^7^ and 2.5 × 10^7^ p/s, respectively) (Fig. 3b). We performed bioimaging analysis to compare vector biodistribution in the different organs. AAV9mut-B, -C, and -D showed a localization pattern similar to that of AAV9wt, with the majority of the signal coming from the upper abdominal cavity and some signal from the head region at day 15 (Fig. 3c) and a widespread signal extended to the thoracic cavity and the hindlimbs at day 35 (Fig. 3c). AAV9mut-A animals displayed a significantly lower luciferase signal localized mainly in the abdominal and thoracic cavities with some expression from the head and the hindlimbs, at both time points (Fig. 3c). We next compared the VGCN in liver, lung, heart, brain, and skeletal muscle (tibialis anterioris) which were collected at sacrifice (Fig. 3a, d). AAV9wt showed a predominant tropism for liver, heart, and lung with less copies found in the brain and skeletal muscle, similarly to what has been previously observed by analyzing vector biodistribution after tail vein injection of AAV9 serotype in mice [41]. In the liver, VGCN was comparable for AAV9wt and AAV9mut-B, while AAV9mut-A and -D displayed a significantly lower liver transduction (Fig. 3d). A slightly higher VGCN in liver was observed for AAV9mut-C (Fig. 3d). A similar trend was observed in the lung, where VGCN was lower in animals injected with AAV9mut-A and higher in animals injected with AAV9mut-C (Fig. 3d). On the other hand, no significant differences between the vectors were observed in terms of VGCN in the heart, brain, and muscle (Fig. 3d). Overall, integrated data from luciferase expression and tissue biodistribution (Fig. 3b–d) showed two variants (AAV9mut-A and -D) displaying lower liver transduction, resulting in less luciferase activity over time, and two variants comparable to AAV9wt (AAV9mut-B and -C), with AAV9mut-C displaying a better liver and lung transduction. At the time of sacrifice, we also collected aortic samples from the mice, in order to analyze VGCN and transgene expression in the vasculature. The group injected with AAV9mut-B showed the highest VGCN in aortic tissue with 0.5 ± 0.1 copies/cell, significantly different from AAV9wt and the other mutants (*p* = 0.01, Fig. 3e). In order to compare transduction efficiency of the different vectors in aortic tissue, we examined the luciferase enzyme activity and we found that AAV9mut-B had the highest expression [17933 ± 3821 relative light units/mg total protein], this level of transduction being three-fold times more than the level observed with AAV9wt and the other mutants (Fig. 3f), data which correlated with the VGCN in aorta. Based on the observations derived from this study, which paralleled the data obtained in vitro (Fig. 2b), we selected the AAV9mut-B as the variant to be further explored for the delivery of VWF transgene. We produced a dual hybrid vector, named ICAM2-VWF, with the previously described endothelial-specific expression cassettes (Fig. 1a) incorporated into the modified AAV9mut-B serotype. In parallel, we produced a liver-specific dual hybrid vector named hAAT-VWF in which the expression cassette was put under the control of the alpha-1 antitrypsin promoter with Apo E enhancer, an hepatocyte-specific regulatory element extensively used by us and others to provide strong transgene expression from the liver [31, 42, 43]. We evaluated the expression of the large VWF transgene in vivo after intravenous injection in VWF deficient mice [20] of either the endothelial-specific ICAM2-VWF or the hepato-specific hAAT-VWF dual vector used as control, at a dose of 5 × 10^11^ vg/mouse of each vector (Fig. 4a). As negative controls, the AAVs expressing VWF 5′ alone were administered (Fig. 4a). Mice treated with ICAM2-VWF or hAAT-VWF stably expressed the transgene over time, showing VWF plasma levels between 1 and 1.5% of those of wt animals (Fig. 4b). On the other hand, the protein was undetectable in control mice injected with the ICAM2- or hAAT-VWF 5′ vector alone (Fig. 4b). Because VWF circulates in a tight complex with the procoagulant FVIII, thus playing an important role in its production, stabilization, function, and immunogenicity [44], we measured FVIII activity (FVIIIa:C) before vector administration and 2 weeks post injection, concomitantly with the first peak of transgene expression. We did not observe any significant increase in both mice injected with ICAM2- or hAAT-VWF, thus indicating that in this experimental condition the residual VWF levels are not sufficient to appreciate changes in FVIIIa:C over time (Fig. 4c and Supplementary Table 1). Interestingly, the usage of a minimal ICAM2 promoter resulted in vivo in VWF expression levels comparable to those achieved with the liver-specific hAAT enhancer–promoter. However, it has to be considered that, when expressed from ECs in physiological conditions, VWF release in circulation is tightly regulated [1] and the protein is stored into specialized secretory granules, which are not present in hepatocytes [45]. Therefore, we cannot exclude differences in terms of total expression from the two promoters since the intracellular VWF levels are likely not to be the same in hepatocytes and ECs. This was suggested also by an in vitro experiment in which HuH7 cells transduced with hAAT-VWF dual vector did not show VWF protein accumulation intracellularly 72 h post transduction (Supplementary Fig. 2). Importantly, in our study, we did not detect protein products other than full-size VWF in vitro by western blot analysis with the usage of a polyclonal antibody. These truncated forms could originate from the 5′ and 3′ VWF which do not undergo a productive genome reassembly [25, 35, 46]. We further confirmed this result in vivo in VWF deficient mice, where we did not detect the presence of truncated proteins from in the animals injected with the 5′ VWF vectors. In conclusion, we developed a capsid-modified AAV9 in order to reconstitute the large VWF gene expression via cell type-specific targeting, providing the first proof of principle that a systemic injection of an endothelial-specific dual AAV vector provide VWF stable expression in VWF deficient mice. A limit of this study was related to the fact that VWF levels obtained in vivo were stable over time but subtherapeutic, as demonstrated by the inability to rescue FVIII activity in this experimental setting. Future studies will be therefore needed to better investigate VWF expression profile from ECs and to further improve the expression cassette (i.e., testing of different recombinogenic regions or enhancers) in order to increase basal expression levels and evaluate the endothelial-derived VWF multimerization profile and hemostatic potential in plasma.

## Supplementary information

Supplementary material
