# Supplementary material for: Development of a dual hybrid AAV vector for endothelial-targeted expression of von Willebrand factor
Source: Gene Ther. 2021 Jan 17;30(3-4):245–54. doi: 10.1038/s41434-020-00218-6 (PMC10113149; doi:10.1038/s41434-020-00218-6)
Supplement: Supplementary file 1 — Supplementary material [file 41434_2020_218_MOESM1_ESM.docx]

**Supplementary Figure 1**

**
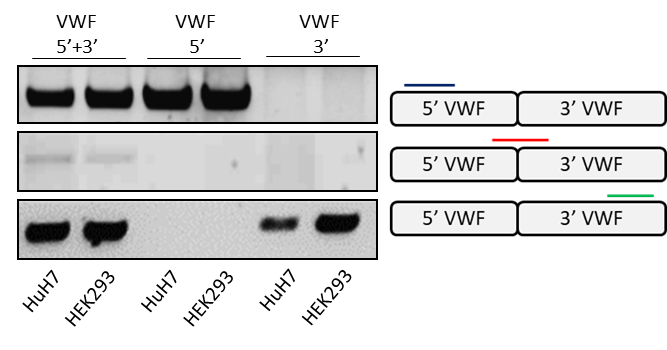
**

PCR amplification of the VWF genomic products in HuH7 and HEK293 cell lysates. On the right, the scheme of the primers used to amplify the VWF 5’, VWF 3’ and the junction region is reported.

**Supplementary Figure 2**

**
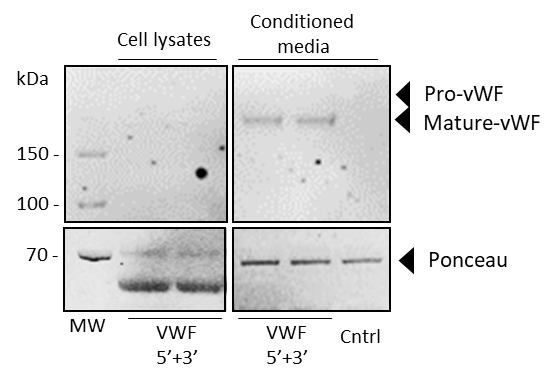
**

Representative western blot analyses on HuH7 cell lysates and conditioned media samples collected 72 h post transduction with the hAAT-VWF dual vector. MW, molecular weight marker. Cntrl, untransduced HuH7 cells.

**Supplementary Table 1**

Table reporting VWF expression levels and FVIII activity (% FVIIIa:C) in plasma over time in single animals treated with either hAAT-VWF or ICAM2-VWF dual vectors. Data are expressed as percentage relative to the average levels of C57Bl6 wt mice (set as 100%).
